# MicroRNA-125a-5p Affects Adipocytes Proliferation, Differentiation and Fatty Acid Composition of Porcine Intramuscular Fat

**DOI:** 10.3390/ijms19020501

**Published:** 2018-02-07

**Authors:** Jingjing Du, Yan Xu, Peiwen Zhang, Xue Zhao, Mailin Gan, Qiang Li, Jideng Ma, Guoqing Tang, Yanzhi Jiang, Jinyong Wang, Xuewei Li, Shunhua Zhang, Li Zhu

**Affiliations:** 1 College of Animal Science and Technology, Sichuan Agricultural University, Chengdu 611130, China; 18111521611@163.com (J.D.); yiyazhi@yahoo.com (Y.X.); sicau_zhangpeiwen@163.com (P.Z.); 18227588896@163.com (X.Z.); 18299095425@139.com (M.G.); jideng_ma@sina.com (J.M.); tyq003@163.com (G.T.); xuewei.li@sicau.edu.cn (X.L.); 2 Sichuan Province General Station of Animal Husbandry, Chengdu 611130, China; 18398635864@163.com; 3 College of Life and Science, Sichuan Agricultural University, Chengdu 611130, China; jiangyz04@163.com; 4 Chongqing Academy of Animal Sciences, Chongqing 402460, China; kingyou@vip.sina.com

**Keywords:** intramuscular fat, fatty acid composition, differentiation, micoRNA-125a-5p, *kruppel like factor 3*

## Abstract

Intramuscular fat (IMF) content and composition are considered crucial indicators of porcine meat quality. However, the molecular mechanism of porcine IMF development is still mostly unclear. Recently, new evidence suggested that microRNA (miRNAs) play important roles in porcine intramuscular adipogenesis. Previously, microRNA-125a-5p (miR-125a-5p) was identified as an important regulator of adipogenesis. In the present study, we found that the expression of miR-125a-5p is dynamically regulated during porcine intramuscular preadipocytes differentiation and that its expression levels in different porcine muscle tissues were negatively involved with IMF content. To investigate the potential function role of miR-125a-5p in IMF development, porcine intramuscular preadipocytes were collected and transfected with miR-125a-5p mimics, inhibitors, or a negative control (NC), respectively. The results showed that overexpression of miR-125a-5p promoted proliferation and inhibited differentiation of porcine intramuscular preadipocytes while inhibition of miR-125a-5p had the opposite effects. Furthermore, a luciferase reporter assay demonstrated that porcine *kruppel like factor 3* (*KLF13*) is a target gene of miR-125a-5p during porcine intramuscular preadipocytes differentiation. Interestingly, porcine *ELOVL fatty acid elongase 6* (*ELOVL6*), a regulator of fatty acid composition, was also identified as a target gene of miR-125a-5p during porcine intramuscular adipogenesis. Further studies show that miR-125a-5p overexpression reduced total saturated fatty acids (SFA) content and monounsaturated fatty acids (MUFA)/SFA ratios while having no significant impact on polyunsaturated fatty acids (PUFA)/SFA and n-6/n-3 ratios. Taken together, our results identified that miR-125a-5p may be a novel regulator of porcine intramuscular adipogenesis and the fatty acid composition of porcine IMF.

## 1. Introduction

Currently, pork production is a still growing industry on the international market [1]. Increasing evidence suggests that pork has become the most consumed meat worldwide accounting for more than 40% of the meat product in the world [1,2,3]. As living standards rise, however, more and more people pay close attention to porcine quality. Intramuscular fat (IMF) content and composition such as fatty acid are important meat quality characteristics, which not only play important roles in the quality of meat including muscle color, tenderness, water-holding capacity, juiciness and flavor of cooked meat, but are also closely associated with human health [4,5,6,7]. The quality of porcine meat is supposed to increase with higher IMF. An IMF content of 2–2.5% were even considered by some researchers as the optimal level in terms of sensory properties of porcine meat [6]. Decreasing dietary saturated fatty acids (SFA), increasing monounsaturated (MUFA) fatty acids, or reducing the polyunsaturated (PUFA) n-6/n-3 fatty acid ratio in meat may reduce the risk of cardiovascular disease and improve human health [8,9,10]. Nevertheless, the molecular mechanism regulating lipid accumulation and fatty acid composition in IMF among pork products remain poorly understood.

MicroRNAs (miRNAs) are an evolutionarily conserved group of endogenous, small non-coding regulatory molecules, which were demonstrated to take part in various biological processes such as cancer, mitochondrial biogenesis, cell proliferation, and differentiation by regulating target genes expression at post-transcriptional levels [11,12,13]. Recently, numerous studies reported that miRNA are involved in intramuscular preadipocyte development including proliferation and differentiation. For instance, Li et al. [14] found that miR-143 inhibited proliferation but promoted differentiation among bovine intramuscular preadipocytes. Guo et al. [15] suggested that up-regulated miR-145 expression inhibited porcine preadipocyte differentiation by negatively regulating *insulin receptor substrate 1* (*IRS1*). Sun et al. [16] demonstrated that miR-34a enhanced adipogenesis in porcine intramuscular preadipocytes through the extracellular regulated MAP kinase (Erk) signaling pathway. Previously, miR-125a-5p was shown to mediate adipogenesis in 3T3-L1 cells and porcine preadipocytes [17,18]. However, whether miR-125a-p is associated with regulation of adipogenesis in IMF of pork meat is still unclear. Here, we show that expression levels of miR-125a-5p in porcine muscle tissues were negatively associated with IMF content. Function analysis demonstrated that miR-125a-5p may promote proliferation but may also inhibit differentiation of porcine intramuscular preadipocytes by regulating *KLF13*. In addition, further studies showed that miR-125a-5p could affect fatty acid composition in porcine intramuscular adipocytes by regulating *ELOVL6*, which suggests that miR-125a-5p may play an important role in porcine intramuscular adipogenesis.

## 2. Results and Discussion

### 2.1. The Expression Levels of miR-125a-5p Was Negatively Associated with Porcine IMF Content

**Figure 1:**
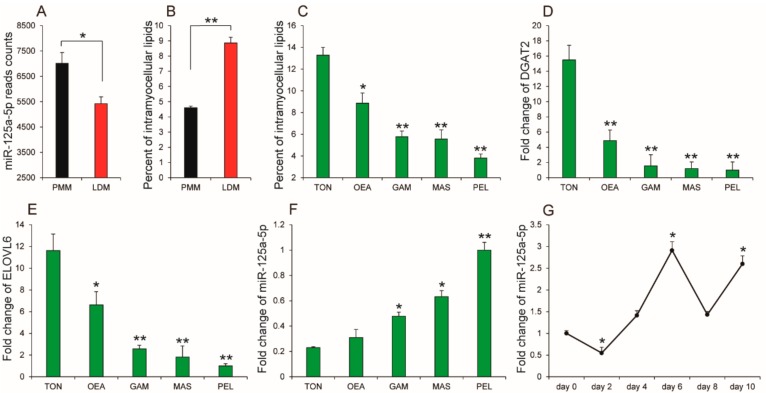
miR-125a-5p was negatively associated with porcine intramuscular fat (IMF) content. (**A**) The relative expression levels of miR-125a-5p in psoas major muscle (PMM) and longissimus dorsi muscle (LDM) (*n* = 3 per sample); (**B**) content of porcine IMF in PMM and LDM (*n* = 3 per sample); (**C**) IMF content in tongue (TON), obliquus externus abdominis (OEA), gastrocnemius muscle (GAM), masseter (MAS), and peroneal longus (PEL) (*n* = 3 per sample); (**D**–**F**) expression levels of *diacylglycerol O-acyltransferase 2* (*DGAT2*), *ELOVL fatty acid elongase 6* (*ELOVL6*) and miR-125a-5p in TON, OEA, GAM, MAS, and PEL (*n* = 3 per sample); (**G**) the relative expression levels of miR-125a-5p during porcine intramuscular preadipocytes differentiation (*n* = 3 per sample per time point). All results are presented as mean ± SEM. * *p* < 0.05; ** *p* < 0.01.

miR-125a-5p has been identified as an important regulator of myogenesis and adipogenesis [18,19,20]. Recently, we found that miR-125a-5p was expressed at higher levels in psoas major muscle (PMM) than the longissimus dorsi muscle (LDM) (Figure 1A), of which PMM has a higher percentage of IMF than LDM [21,22] (Figure 1B). In order to investigate whether miR-125a-5p is involved in the formation of porcine intramuscular fat (IMF), we performed quantitative real-time PCR (qRT-PCR) to compare the expression levels of miR-125a-5p in different muscles from the Liangshan pig. The results show that the IMF content of tongue muscle (TON) and obliquus externus abdominis (OEA) is significantly greater than other muscles including gastrocnemius muscle (GAM), masseter (MAS), and peroneal longus (PEL) (Figure 1C). Previous studies indicated that *diacylglycerol O-acyltransferase 2* (*DGAT2*) and *ELOVL fatty acid elongase 6* (*ELOVL6*) are positively correlated with IMF in pork meat [23,24]. Consistent with that, the expression levels of *DGAT2* and *ELOVL6* showed a similar varying tendency with the content of IMF in the above muscle tissues (Figure 1D,E). Interestingly, qRT-PCR analysis showed that miR-125a-5p was expressed at higher levels in both PEL and MAS when compared to both TON and OEA (Figure 1F), suggesting that miR-125a-5p expression might be closely associated with IMF content. In order to further reveal the relationship between miR-125a-5p and IMF, we isolated porcine intramuscular preadipocytes to induce differentiation and then evaluated the temporal patterns of miR-125a-5p expression during porcine intramuscular adipogenesis. As shown in Figure 1G, the expression levels of miR-125a-5p gradually increased up to day 6 of intramuscular preadipocytes differentiation but then slightly decreased after that. This result showed a similar expression pattern with miR-425-5p, which have been demonstrated to inhibit differentiation and proliferation in porcine intramuscular preadipocytes [25]. Overall, these results suggest that miR-125a-5p may be negatively associated with porcine intramuscular adipogenesis.

### 2.2. miR-125a-5p Promoted Proliferation of Porcine Intramuscular Preadipocytes

**Figure 2:**
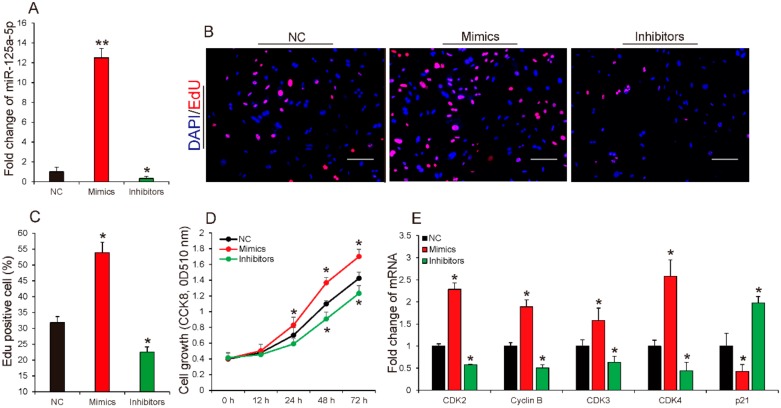
miR-125a-5p promoted proliferation of porcine intramuscular preadipocytes. (**A**) Relative expression levels of miR-125a-5p in porcine intramuscular preadipocytes transfected with miR-125a-5p mimics inhibitors or negative control (NC) (*n* = 3 per treatment); (**B**,**C**) 5-ethynyl-2′-deoxyuridine (EdU) proliferation and (**D**) Cell Counting kit 8 (CCK-8) assays were performed to determine cell proliferation (*n* = 6 per treatment per time point); (**E**) the relative expression levels of *cyclin-dependent kinases 2* (*CDK2*), *Cell cycle protein B* (*Cyclin B*), c*yclin-dependent kinases 3* (*CDK3*), c*yclin-dependent kinases 4* (*CDK4*), and *cyclin-dependent kinase inhibitor 1A* (*p21*) when porcine intramuscular preadipocytes were transfected with mimics, inhibitors, or NC for 2 days (*n* = 3 per treatment). Scale bar, 100 μm. All results are presented as mean ± SEM. * *p* < 0.05; ** *p* < 0.01.

Preadipocytes proliferation and differentiation are the basis on adipogenesis. To elucidate the potential function of miR-125a-5p in porcine intramuscular adipogenesis, we first explored the effect of miR-125a-5p on porcine intramuscular preadipocyte proliferation. As shown in Figure 2A, synthetic miR-125a-5p mimics inhibitors and the negative control (NC), which were transfected into porcine intramuscular preadipocytes, respectively. Additionally, miR-125a-5p mimics significantly increased the expression levels of miR-125a-5p by 13-fold in preadipocytes while endogenic expression of miR-125a-5p in preadipocytes was remarkably inhibited by transfecting with miR-125a-5p inhibitors. Subsequently, 5-ethynyl-2′-deoxyuridine (EdU) and Cell Counting kit 8 (CCK-8) analysis procedures were performed to evaluate the effect of miR-125a-5p on proliferation of porcine intramuscular preadipocytes. As shown in Figure 2B,C, EdU analysis suggested that, compared to the control group, miR-125a-5p overexpression significantly increased the ratio of EdU positive preadipocytes while inhibition of miR125a-5p strongly decreased the ratio of EdU positive preadipocytes. Meanwhile, CCK8 detection confirmed this effect, which showed that transfection miR-125a-5p mimics remarkably increased the total number of preadipocytes when compared with the control group. In contrast, the total number of preadipocytes transfected with miR-125a-5p inhibitors were significantly reduced compared with the control group (Figure 2D). These findings indicated that miR-125a-5p may promote porcine intramuscular preadipocyte proliferation.

Cyclin-dependent kinases (CDKs) such as *cyclin-dependent kinases 2* (*CDK2*) and c*yclin-dependent kinases 3* (*CDK3*) have been recognized as key regulators of cell growth and proliferation in eukaryotes, which are required for G1-to-S phase transition in mammalian cells [26,27]. *Cell cycle protein B* (*Cyclin B*) is necessary for the progression of the cells into and out of the M phase of the cell cycle [28]. Conversely, overexpression of *cyclin-dependent kinase inhibitor 1A* (*p21*) may inhibit cell proliferation by causing cell cycle arrest, which is an inhibitor of CDKs [29]. To confirm whether miR-125a-5p mediation of the porcine intramuscular preadipocyte proliferation is associated with these factors, we compared gene expression in cells transfected, respectively, with miR-125a-5p mimics, inhibitors, or NC. As shown in Figure 2E, qRT-PCR analysis illustrated that overexpression of miR-125a-5p significantly up-regulated the expression levels of *CDK2*, *CDK3*, *CDK4* and *Cyclin B*, and inhibition of miR-125a-5p strongly down-regulated the expression of these genes. Additionally, overexpression or inhibition of miR-125a-5p caused a decrease or an increase in the expression of *p21* in porcine intramuscular preadipocytes, respectively. Taken together, all results suggest that miR-125a-5p might inhibit proliferation of porcine intramuscular preadipocytes.

### 2.3. miR-125a-5p Inhibited Differentiation of Porcine Intramuscular Preadipocytes by Directly Targeting KLF13

**Figure 3:**
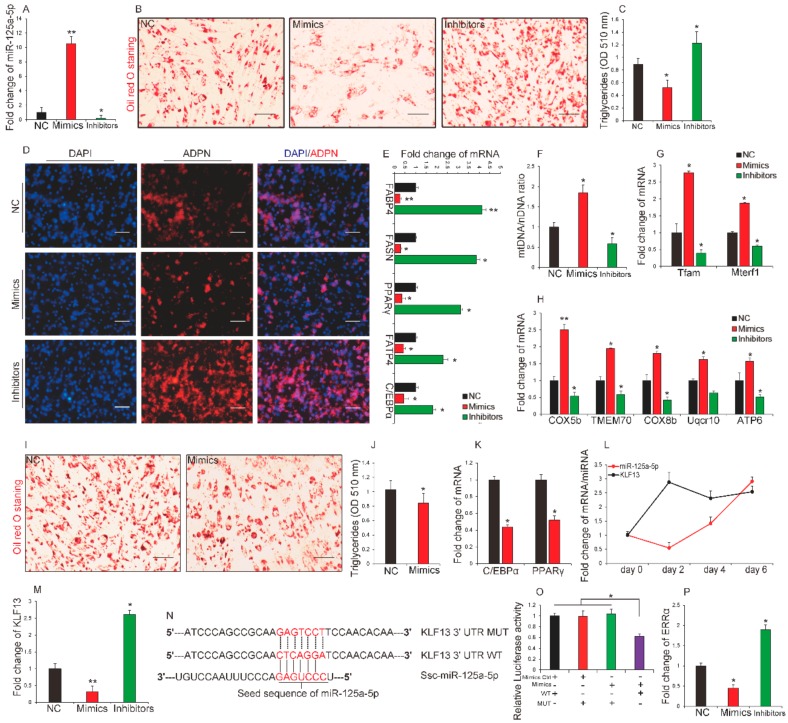
miR-125a-5p inhibited differentiation of porcine intramuscular preadipocytes by directly targeting *Kruppel-like factor 13* (*KLF13*). After porcine intramuscular preadipocytes were induced differentiate and transfected with miR-125a-5p mimics, inhibitors or negative control (NC) for 8 days, (**A**) the relative expression levels of miR-125a-5p were measured by qRT-PCR (*n* = 3 per treatment); (**B**) Cells were stained with oil red O (*n* = 3 per treatment); (**C**) triglycerides content was measured by spectrophotometric analysis (*n* = 4 per treatment); (**D**) immunofluorescence of adiponectin was performed (*n* = 3 per treatment); (**E**) The relative expression levels of genes related to adipocytes differentiation (*n* = 3 per treatment). Additionally, porcine intramuscular preadipocytes that were induced differentiated for 4 days and were transfected respectively with miR-125a-5p mimics, inhibitors, or NC. (**F**) Mitochondrial content were evaluated by measuring the ratio of mtDNA:nDNA (*n* = 3 per treatment); The relative expression levels of genes related to (**G**) mitochondrial biogenesis and (**H**) mitochondrial energy metabolism (*n* = 3 per treatment); (**I**) cells were stained with oil red O (*n* = 3 per treatment); (**J**) triglycerides content was measured by spectrophotometric analysis (*n* = 3 per treatment); (**K**) the relative expression levels of *CCAAT/enhancer binding protein α* (*C/EBPα*), and *peroxisome proliferator activated receptor γ* (*PPARγ*) (*n* = 3 per treatment); (**L**) The relative expression levels of miR-125a-5p and *KLF13* during differentiation of porcine intramuscular preadipocytes (*n* = 3 per treatment per time point); (**M**) The expression levels of *KLF13* after cells were transfected with mimics, inhibitors, or NC (*n* = 3 per treatment); (**N**) sequence alignment of Ssc-miR-125a-5p with 3′-UTR of porcine *KLF13* mRNA; (**O**) Luciferase assays revealed the repressive effect of miR-125a-5p on the activity of *KLF13* (*n* = 7 per treatment); (**P**) the expression levels of *estrogen related receptor α* (*ERRα*) after cells were transfected with mimics, inhibitors or NC (*n* = 3 per treatment). Scale bar, 100 μm. All results are presented as mean ± SEM. * *p* < 0.05; ** *p* < 0.01.

Next we identified the functional role of miR-125a-5p during porcine intramuscular preadipocytes differentiation. Transfection efficiency is shown in Figure 3A. We observed that overexpression of miR-125a-5p significantly inhibited intramuscular preadipocyte differentiation as determined by measuring the Oil Red O staining signal (Figure 3B) and triglycerides (TG) contents (Figure 3C) at day 8 of preadipocyte differentiation. By contrast, inhibition of miR-125a-5p had an opposite effect on the differentiation of porcine intramuscular preadipocytes compared with miR-125a-5p overexpression. In addition, by performing immunofluorescence of adiponectin and qRT-PCR analysis, we observed that transfection of miR-125a-5p mimics for 8 days significantly repressed the expression levels of adipogenic marker including adiponectin, *CCAAT/enhancer binding protein α* (*C/EBPα*), *peroxisome proliferator activated receptor γ* (*PPARγ*), *adipocyte fatty acid-binding protein 4* (*FABP4*), and *fatty acid synthase* (*FASN*) while transfection of miR-125a-5p inhibitors remarkably promoted the expression of these factors when compared with the control group (Figure 3D,E). These results suggest that miR-125a-5p may play a negatively functional role during porcine intramuscular preadipocyte differentiation. Lipid metabolism is a continuum from emulsification and uptake of lipids in the intestine to cellular uptake and transport to compartments such as mitochondria [30]. Increasing mitochondrial biogenesis in white adipose tissues can impair white adipocyte formation and enhance energy output in the form of heat [31,32]. Recently, Maude et al. [32] reported that miR-125b could affect mitochondrial biogenesis. These findings gave us a hypothesis that miR-125a-5p may accelerate the expenditure of porcine intramuscular adipocytes by increasing mitochondrial biogenesis. To test this hypothesis, porcine intramuscular preadipocytes were induced to differentiate for four days and were then transfected, respectively, with miR-125a-5p mimics, inhibitors, or NC. After four days of transfection, we measured mitochondrial content by calculating the ratio of mitochondrial DNA:nuclear DNA (mtDNA:nDNA). As shown in Figure 3F, overexpression of miR-125a-5p promoted mitochondrial biogenesis in adipocytes while inhibition of miR-125a-5p suppressed mitochondrial biogenesis in porcine intramuscular adipocytes when compared with the control group. Consistent with changes in mitochondrial biogenesis, genes related to mitochondrial biogenesis (*Mterf1*, *Mitochondrial transcription termination factor 1*; *Tfam*, *Transcription factor A*) (Figure 3G) and mitochondrial energy metabolism (*Cox5b*, *Cytochrome c oxidase subunit Vb*; *TMEM70*, *Transmembrane protein 70*; *Cox8b*, *Cytochrome c oxidase subunit VIIIb*; *Uqcr10*, *Ubiquinol-cytochrome c reductase*, *complex III subunit X*; and *ATP6*, *ATP synthase F0 subunit 6*) (Figure 3H) were expressed at higher or lower levels in adipocytes transfected with miR-125a-5p mimics or inhibitors, respectively. In addition, along with the increasing or decreasing of mitochondria, transfection of miR-125a-5p mimics significantly decreased the number of oil red O^+^ cells (Figure 3I), triglyceride content (Figure 3J), and expression levels of two key transcriptional regulators of adipogenesis called C/EBPα and PPARγ (Figure 3K). The findings suggest that miR-125a-5p may accelerate IMF expenditure in pork products by enhancing mitochondrial biogenesis and energy metabolism. Taken together, these results suggest that miR-125a-5p could inhibited differentiation of porcine intramuscular preadipocytes.

Previously, several studies demonstrated that miRNAs take part in many biological processes by negatively regulating target genes through interaction with the 3′-untranslated regions (3′-UTR) of the target mRNAs [33,34,35]. To further explore detailed molecular mechanisms that miR-125a-5p inhibits porcine intramuscular preadipocytes differentiation, we predicted target genes of miR-125a-5p using TargetScan 7.1. Among the potential target genes, we observed that the 3′-UTR region of *Kruppel-like factor 13* (*KLF13*) mRNA had a binding site for miR-125a-5p seed sequences and could also accelerate porcine adipocyte differentiation [36]. In addition, miR-125a-5p and *KLF13* were reciprocally expressed during porcine intramuscular preadipocytes differentiation (Figure 3L). Overexpression or inhibition of miR-125a-5p downregulated or upregulated the expression of *KLF13*, respectively (Figure 3M). These findings lead us to hypothesize that *KLF13* is a direct target of miR-125a-5p for negatively regulating differentiation of porcine intramuscular preadipocytes. To confirm that, luciferase reporter assays were performed. As shown in Figure 3N,O, we observed that transfection of miR-125a-5p mimics in porcine intramuscular preadipocytes directly repressed the luciferase activities of the wild-type *KLF13* 3′-UTR (WT-*KLF13*) reporter when compared to the control group while mutation of the miR-125a-5p binding site in porcine *KLF13* 3′-UTR completely stopped this response. This suggests that *KLF13* is a direct target gene of miR-125a-5p during differentiation of porcine intramuscular preadipocytes. Additionally, Ji et al. [18] suggested that *estrogen related receptor α* (*ERRα*) is a directly target gene of miR-125a-5p inhibiting porcine preadipocytes differentiation. We also found that miR-125a-5p overexpression significantly repressed *ERRα* while inhibition of miR-125a-5p enhanced its expression during differentiation of porcine intramuscular preadipocytes (Figure 3P). In summary, the data revealed that miR-125a-5p inhibited differentiation of porcine intramuscular preadipocytes by negatively regulating *KLF13*.

### 2.4. miR-125a-5p Affected Fatty Acid Composition in Porcine Intramuscular Adipocytes

**Figure 4:**
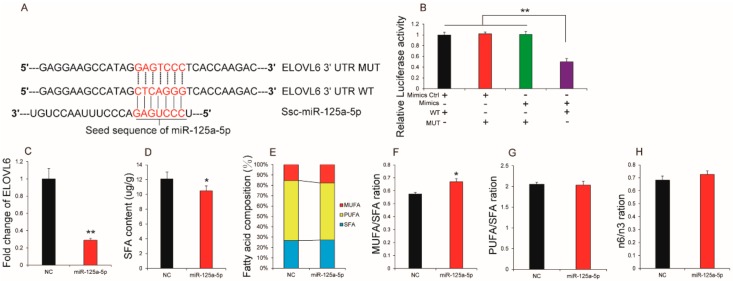
miR-125a-5p affected fatty acid composition in porcine intramuscular adipocytes. (**A**) Sequence alignment of Ssc-miR-125a-5p with 3′-UTR of porcine *ELOVL6* mRNA; (**B**) Luciferase assays revealed the repressive effect of miR-125a-5p on the activity of *ELOVL6* (*n* = 7 per treatment). After porcine intramuscular adipocytes were transfected with mimics or NC; (**C**) the expression levels of *ELOVL6* (*n* = 3 per treatment); (**D**) Saturated fatty acids (SFA) content in mimics group and control group (*n* = 3 per treatment); (**E**) Fatty acid composition difference (*n* = 3 per treatment); (**F**) monounsaturated fatty acids (MUFA)/SFA ration (*n* = 3 per treatment); (**G**) polyunsaturated fatty acids (PUFA)/SFA ration (*n* = 3 per treatment); (**H**) n-6/n-3 ration (*n* = 3 per treatment). All results are presented as mean ± SEM. *n* = 3. * *p* < 0.05; ** *p* < 0.01.

Fatty acids are major contributors of meat nutritional value, which is associated with human health [37,38]. By performing luciferase reporter assays, we identified that porcine *ELOVL6* is a target gene of miR-125a-5p, which has been shown to be a major regulator of fatty acid composition in pig tissue (Figure 4A,B). We then measured the difference in fatty acid composition after porcine intramuscular preadipocytes were induced to differentiate and transfected with miR-125a-5p mimics or negative control for eight days. As shown in Table 1, we found that miR-125a-5p overexpression had negative influence on C14:0, C15:0, C16:0, C16:1, C18:0, C18:1n9c, C20:2, C20:3n3, C20:4n6, C20:5n3, and significantly increased C17:1 and C20:1. However, no significant difference between the mimics group and the control group was observed for C6:0, C8:0, C10:0, C12:0, C17:0, C18:2n6c, C18:3n6, C20:0, C20:3n3, C20:3n6 and C22:6. Notably, these changes in content of C18:0 and C18:1 (including C18:1n9t and C18:1n9c) between the mimics group and the negative group were consistent with prior results that miR-125a-5p overexpression significantly downregulated *ELOVL6* expression in porcine intramuscular adipocytes (Figure 4C). Previous studies demonstrated that *ELOVL6* could elongate C12-C16 to longer fatty acids and play an important role in the synthesis of stearic acid (C18:0) and oleic acid (C18:1) [39,40]. Meat is a primary source of the total amount of saturated fatty acids (SFA), which has been identified as a dietary risk factor closely associated with coronary heart disease and even various cancers [41,42]. Reducing the intake of SFA and increasing the intake of monounsaturated fatty acids (MUFA) can improve human health [9,10]. Further analysis found that miR-125a-5p overexpression significantly reduced total SFA content when compared with the control group (Figure 4D). However, overexpression had little impact on MUFA, SFA, and polyunsaturated fatty acids (PUFA) proportion in total fatty acids (Figure 4E). Furthermore, decreasing SFA content in mimics group when compared to the control group lowered levels of C14:0 and C18:0, which have a cholesterol rising effect [43] found in the mimics group but not the control group (Table 1). This suggests that miR-125a expression levels in porcine intramuscular adipocytes may be related to pork quality.

Additionally, compared to the content of particular fatty acids, nutritionists pay more attention to the PUFA/SFA ratio, the ratio of n-6/n-3, and meat products with higher ratios of PUFA and MUFA relative to SFA. A favorable balance between n-6 and n-3 in PUFA has a beneficial effect on disease prevention [44,45]. In order to further shed light on the relationship between miR-125a-5p and pork quality, we compared the ratios of PUFA/SFA, MUFA/ SFA, and n-6/n-3. The MUFA/SFA ratios were greater in the mimics group than the control group. However, no significant difference in PUFA/SFA and n-6/n-3 ratios was observed between the mimics group and the control group (Figure 4F–H). Taken together, these results suggest that miR-125a-5p could affect fatty acid composition in porcine intramuscular adipocytes.

## 3. Materials and Methods

### 3.1. Muscle Samples Collection

Seven female LiangShan pigs that were reared in the same environment and fed the same diet were raised from birth to 180 days old. Subsequently, three pigs at a bodyweight 50–65 kg were selected for slaughter using the same strategy. In order to measure intramuscular fat (IMF) content and gene expression, muscle tissues including tongue muscle (TON), obliquus externus abdominis (OEA), gastrocnemius muscle (GAM), masseter (MAS), and peroneal longus (PEL) were quickly collected and stored at −20 °C or −80 °C, respectively. All experimental procedures were performed according to the guide for Animal Care and Ethics Committee of Sichuan Agricultural University, Sichuan (permit number DKY-S20143135, June 2014.).

### 3.2. Porcine Intramuscular Preadipocytess Isolation and Culture

According to previous reports [25,46,47], one female LiangShan Pig was sacrificed for porcine intramuscular preadipocytess isolation at three days. Briefly, Longissimus dorsi muscles (LDM) were aseptically isolated and washed three times using phosphate buffered saline (PBS, Gibco, Grand Island, NY, USA) containing 200 U/mL penicillin–streptomycin (Hyclone, Logan, UT, USA). After all visible connective tissue was removed, the isolated muscle tissue was finely minced and then digested in serum free Dulbecco’s modified eagle medium/F12 (DMEM/F1, Hyclone) with 0.2% type-II collagenase (Invitrogen, Carlsbad, CA, USA) in a shaking water bath with 40 rpm speeds for 2 h at 37 °C. The digest sample was filtered aseptically through 70 and 200 μm steel mesh filters to isolate digested cells. Then the filtered cells were rinsed three times with serum free DMEM/F12 medium and then centrifuged twice at 1500× *g* for 10 min. Subsequently, cells were seeded at a density 6 × 10^5^ per 60 mm culture dish in DMEM/F12 medium with 10% fetal bovine (FBS, Gibco) and penicillin–streptomycin and cultured at 37 °C with 5% CO_2_. After 1 h, cells were washed with DMEM/F12 medium to wipe off un-adhered cells. After reaching 80% confluence, cells were digested with 0.05% trypsin and plated at a density of 5 × 10^4^ cells/cm in a 6-well plate for further research.

To induce differentiation, the above cells were cultured in induction medium containing DMEM/F12, 10% FBS, 100 U/mL penicillin–streptomycin, 1 nM dexamethasone (DEX, Sigma, St. Louis, MO, USA), 5 ng/mL insulin (Sigma), and 0.5 mM 3-isobutyl 1-methylxanthine (IBMX, Sigma). After two days, the medium was changed with DMEM/F12 containing 10% FBS, 100 U/mL penicillin–streptomycin, and 5 ng/mL insulin. The medium was changed every two days until day 8.

### 3.3. Cells Transfection

To evaluate the effect of miR-125a-5p on the proliferation and differentiation of porcine intramuscular preadipocytes, cells were transfected respectively with synthetic miR-125a-5p mimics (sequence: 5′-UCCCUGAGACCCUUUAACCUGU-3′), inhibitors (sequence: 5′-ACAGGUUAAAGGGUCUCAGGGA-3′), or negative control (NC, sequence: 5′-UUUGUACUACACAAAAGUACUG-3′, 5′-CAGUACUUUUGUGUAGUACAAA-3′) (all purchased from RIBOBIO, Guangzhou, China). For proliferation, when the density of cells in 96-well plates reached 40%, miR-125a-5p mimics (50 nM), inhibitors (100 nM), or NC (50 nM) were transfected into cells using Lipofectamine2000 (Invitrogen) and Opti-MEM (Gibco) culture medium, according to the manufacturer’s instructions. For differentiation, when the density of cells in 12-well plates reached 80%, using the same way, miR-125a-5p mimics, inhibitors, or NC were transfected into cells according to the manufacturer’s instructions, and the culture medium was changed to differentiation medium after 24 h of transfection.

### 3.4. Proliferation Analysis by CCK8 and EdU Assays

Porcine intramuscular preadipocytes seeded in 96-well plates in the DMEM/F12 medium supplement with 10% FBS were transfected with miR-125a-5p mimics, inhibitors, and NC. Cell proliferation was measured at 0, 12, 24, 48, and 72 h of transfection by using the Cell Counting kit 8 (CCK-8, Beyotime, Shanghai, China) according to the manufacturer’s protocol. For 5-ethynyl-2′-deoxyuridine analysis (EdU, RIBOBIO, Guangzhou, China), cells were treated with 10 μM EdU for 24 h after transfection and incubated for a further 10 h. EdU staining was performed according to the manufacturer’s protocol. Images were captured using an Olympus IX53 microscope (Olympus, Tokyo, Japan).

### 3.5. Oil Red O Staining and Triglyceride Analysis

Cells treated for 8 days were washed three times with PBS and fixed in 4% paraformaldehyde solution and incubated with 0.5% Oil Red O for 1 h. After cell samples were further washed three times with PBS, images were captured using an Olympus IX53 microscope (Olympus). To measure triglyceride (TG) contents, stained cells were eluted with isopropanol for 20 min and the optical density (OD) values were detected with a spectrophotometer at a wavelength of 510 nm, as described by our previous report [22,23].

### 3.6. Immunocytochemical Analysis

As described by Du et al. [22], cell samples were washed three times with PBS, fixed in 4% paraformaldehyde for 30 min, and then permeabilized with 0.5% Triton X-100 prior to blocking in 2% goat serum. Subsequently, the above cell samples were incubated with an anti-adiponectin antibody (Bioss, Beijing, China, catalog number: bs-0471R) at 4 °C for 24 h. This was followed by fluorescent secondary antibodies at 37 °C for 1 h. Images were captured using an Olympus IX53 microscope (Olympus).

### 3.7. RNA Extraction and qRT-PCR

Total RNA from cells and muscle tissues were extracted using a TRIzol Reagent (Invitrogen) according to the manufacturer’s instructions. The RNA quality and concentration were estimated using denatured gel electrophoresis and a spectrophotometer (Thermo, Waltham, MA, USA), respectively. Subsequently, cDNA was synthesized and quantitative real-time PCR (qRT-PCR) was performed by the SYBR Premix Ex Taq kit (TaKaRa; LianDa, China) on a CFX96 system (Bio-Rad, Hercules, CA, USA). Relative expression levels of mRNAs and microRNAs were calculated using the 2^−ΔΔ*C*t^ method [48]. The primer sequences used for qRT-PCR are listed in Supplementary Table S1. U6 and β-actin were used as endogenous control genes for miRNA and mRNA, respectively.

### 3.8. Mitochondrial Content

The relative number of mitochondria was determined by measuring the ratio of mtDNA:nDNA as previously described [49]. The relative levels of mtDNA and nDNA were quantified using primers specific for mitochondrial *cytochrome c oxidase subunit 1* (*Cox1*) and the nuclear gene *glucagon* (*GCG*).

### 3.9. Luciferase Reporter Assay

The wild-type 3′UTR of *KLF13* and *ELOVL6* (WT-KLF13 and WT-ELOVL6), Mutant-type 3′UTR of *KLF13*, and *ELOVL6* (Mut-KLF13 and Mut-ELOVL6) were cloned into psiCHECKTM-2 vector at the 3′-end of the *Renilla* gene, which was made by a manufacturer (TsingKe Biotech, Chengdu, China). For luciferase reporter analysis, wild-type 3′UTR or mutant-type 3′UTR were co-transfected respectively with miR-125a-5p mimics, inhibitors, or NC into porcine intramuscular preadipocytess using Lipofectamine3000 (Invitrogen). Cells were harvested at 48 h post-transfection and luciferase activities were measured with the Dual-Glo Luciferase Assay System (Promega, Madison, WI, USA) following the manufacturer’s instructions.

### 3.10. Measurement of IMF and Fatty Acid Composition

The above muscle tissues stored at −20 °C were studied in triplicate (each sample of 3 g) and then IMF measurement was performed and determined as the percentage of fat extracted 3 g of fresh tissues using the Soxhlet petroleum-ether extraction method, as described by a previous study [50]. Furthermore, fatty acids were analyzed according to previously published protocols [51]. After porcine intramuscular preadipocytess were induced, differentiated, and transfected with miR-125a-5 mimics or NC for 8 days, 50 mg cells samples were collected to determine fatty acids composition, which was completed by the Beijing Municipal Science and Technology limited company.

### 3.11. Statistical Analysis

All data are expressed as means ± SE, and statistical analyses were performed using SPSS22.0 software (SPSS22.0, SPSS Science, Chicago, IL, USA). Differences between groups were analyzed by applying Student’s two-tailed *t*-test for two parametric groups and one-way analysis for the three least parametric groups. A value of *p* < 0.05 indicated a significant difference.

## 4. Conclusions

In the present study, we identified a novel epigenetic regulator of porcine intramuscular fat content and composition. The results showed that expression levels of miR-125a-5p in porcine muscle tissues were negatively associated with IMF content, overexpression, or inhibition of miR-125a-5p along with promoted or repressed proliferation of porcine intramuscular preadipocytes, respectively. By directly targeting KLF13, miR-125a-5p inhibited the differentiation of porcine intramuscular preadipocytes. Particularly, we found that miR-125a-5p affected fatty acid composition by negatively regulating ELOVL6, a regulator of fatty acid composition. The data indicated miR-125a-5p may play an import role in porcine intramuscular adipogenesis and fatty acid composition regulation in porcine IMF.

## Figures and Tables

**Table 1:** miR-125a-5p regulate fatty acid composition in porcine intramuscular adipocytes.

Fatty Acid	NC	Mimics	Up/Down
C6:0	0.3489	0.3492	down ^NS^
C8:0	0.5118	0.5119	down ^NS^
C10:0	0.4812	0.4818	down ^NS^
C12:0	0.7860	0.7838	down ^NS^
C13:0	0.0000	0.0000	-
C14:0	1.0003	0.8013	down *
C15:0	0.4443	0.3914	down *
C15:1	0.0000	0.0000	-
C16:0	3.0437	2.3119	down *
C16:1	2.2056	2.0297	down *
C17:0	0.4456	0.4022	down ^NS^
C17:1	0.5076	0.7660	up *
C18:0	4.1666	3.5791	down *
C18:1n9t	0.0000	0.0000	-
C18:1n9c	3.9273	3.6161	down *
C18:2n6t	0.0000	0.0000	-
C18:2n6c	1.2904	1.0772	down ^NS^
C18:3n3	0.0000	0.0000	-
C18:3n6	1.1420	1.0054	down ^NS^
C20:0	0.8700	0.8703	up ^NS^
C20:2	1.3139	0.9447	down *
C20:1	0.3175	0.6081	up *
C20:3n3	0.7617	0.6041	down*
C20:3n6	1.1683	1.1799	up ^NS^
C20:4n6	4.5844	3.9889	down *
C20:5n3	11.5488	8.9343	down **
C21:0	0.0000	0.0000	-
C22:0	0.0000	0.0000	-
C22:1n9	0.0000	0.0000	-
C22:2n6	0.0000	0.0000	-
C22:6	3.3151	3.5985	up ^NS^
C24:0	0.0000	0.0000	-
C24:1	0.0000	0.0000	-
SFA	12.0983	10.4831	down *
PUFA	26.3246	21.3331	down *
MUFA	6.9580	6.8200	down ^NS^
n-6	8.3851	7.1514	down *
n-3	12.3105	9.8584	down *

Saturated fatty acid (SFA) = C8:0 + C10:0 + C12:0 + C14:0 + C15:0 + C16:0 + C17:0 + C18:0 + C20:0 + C22:0; Monounsaturated fatty acid (MUFA) = C14:1 + C16:1 + C18:1 + C20:1 + C24:1; Polyunsaturated fatty acid (PUFA) = C18:2 + C18:3 + C20:2 + C20:3 + C20:4 + C20:5 + C22:2 + C22:6; n-3 = C18:3n3 + C20:3n3 + C20:5n3; n-6 = C18:2n6t + C18:2n6c + C18:3n6 + C20:3n6 + C20:4n6 + C22:2n6. * *p* < 0.05; ** *p* < 0.01; NS, no significant difference.

## Supplementary Materials

Supplementary materials can be found at http://www.mdpi.com/1422-0067/19/2/501/s1.

## Abbreviations

*CDK2*	*Cyclin-dependent kinase 2*
*Cyclin B*	*Cell cycle protein B*
*CDK3*	*Cyclin-dependent kinases 3*
*CDK4*	*Cyclin-dependent kinases 4*
*p21*	*Cyclin-dependent kinase inhibitor 1*
*FABP4*	*Adipocyte fatty acid-binding protein 4*
*FASN*	*Fatty acid synthase*
*PPARγ*	*Peroxisome proliferator activated receptor γ*
*FATP4*	*Fatty acid transporter member 4*
*C/EBPα*	*CCAAT/enhancer binding protein α*
*Tfam*	*Transcription factor A*
*Mterf1*	*Mitochondrial transcription termination factor 1*
*COX5b*	*Cytochrome c oxidase subunit Vb*
*TMEM70*	*Transmembrane protein 70*
*COX8b*	*Cytochrome c oxidase subunit VIIIb*
*Uqcr10*	*Ubiquinol-cytochrome c reductase, complex III subunit X*
*ATP6*	*ATP synthase F0 subunit 6*
*DGAT2*	*Diacylglycerol O-acyltransferase 2*
*ELOVL6*	*ELOVL fatty acid elongase 6*
*KLF13*	*Kruppel-like factor 13*
miRNA	microRNA
IMF	Intramuscular fat content
TON	Tongue
OEA	Obliquus externus abdominis
GAM	Gastrocnemius muscle
MAS	Masseter
PEL	Peroneal longus
qRT-PCR	Quantitative real-time polymerase chain reaction
