# Supplementary material for: MicroRNA-125a-5p Affects Adipocytes Proliferation, Differentiation and Fatty Acid Composition of Porcine Intramuscular Fat
Source: Int J Mol Sci. 2018 Feb 7;19(2):501. doi: 10.3390/ijms19020501 (PMC5855723; doi:10.3390/ijms19020501)
Supplement: Supplementary file 1 [file ijms-19-00501-s001.pdf]

Table S1. The primer sequences used for qRT:PCR. F: forward, R: reverse. U6 and β-actin were used as endogenous control genes for miRNA and mRNA, respectively.

Gene	Primer sequence (5`→3`)
DGAT2	F: GAAAGCGGCTACAGGTCATCTC<br>R: GGGCGTGTTCCAGTCAAA
ELOVL6	F: AGCAGTTCAACGAGAACGAAGCC<br>R:TGCCGACCGCCAAAGATAAAG
FABP4	F: ACAGGAAAGTCAAGAGCACCA<br>R: TCGGGACAATACATCCAACAG
PPARγ	F: CATTCGCATCTTTCAGGG<br>R: GGACGCCATACTTTAGGA
FASN	F: CGTGGGCTACAGCATGATAG<br>R: GAGGAGCAGGCCGTGTCTAT
β-actin	F: TCTGGCACCACACCTTCT<br>R: TGATCTGGGTCATCTTCTCAC
FATP4	F: CCATCTTATTCGCTGCCACC<br>R: AGGCCCACGAACTCATTACA
C/EBPα	F: ACTGCTACAAAGACCCCTCC<br>R: TGTTCGCCAGCTTGAGTTTC
COX1	F: ACTACTGACAGACCGCAACC<br>R: TCCAATGGACATTATGGCTC
GCG	F: GAATCAACACCATCGGTCAAAT<br>R: CTCCACCCATAGAATGCCCAGT
Tfam	F: CTCCTCCTTCGTCGTAGTCC<br>R: ACCCGTAGACAAAGCACTGA
Mterf1	F: CATGACACGCACACCTGAAA<br>R: CGACAAAGGCATTTACGGGT
ATP6	F: TATTTGCCTCTTTCATTGCCC<br>R: GGATCGAGATTGTGCGGTTAT
COX5b	F: TAGTGGGCTGCATCTGTGAA<br>R: CAGCGCATCTTGAGTGTGTT
TMEM70	F: ATCCAGCGCTCTTTCCAAAC<br>R: CACAAGAGCATGCCCAAGTT
Cox8b	F: GGACATTCAGGGTGCCTCTT<br>R: GAAGTGGGAGTTTTGGCTGG
Uqcr10	F: TGGGCGCTCTATTCTTCGAG<br>R: GTCCATGGAGAGTGACGTGA
KLF13	F: CCCCAGAGAAAGCACAAGTG<br>R: AGCTGAACTTCTTCTCGCCT
ERRα	F: CTGGAGCGTGAGGAGTATGT<br>R: AGAAATGGGCCAGCACTTTG

CDK2	F: AGTCTTCTTGCCTGTCCCTC<br>R: CCACTCAGTCACAGCCCTAA
CDK3	F: TGGCCCTCAAGAAGATCAGG<br>R: TTCAGGTCTCGGTGGATGAC
CDK4	F: CGAGAGACCAAAGTGACCCT<br>R: TTGACTGTCCCACCACTTGT
Cyclin B	F: TTGTGTGCCCAAGAAGATGC<br>R: CTCCGAAGAAAATGCAGGGG
P21	F: CTGTGAACTTTAGGGTGCGG<br>R: GGCAGTAAAACCTCGCTAGC
U6	F: GCTTCGAGGCAGGTTACATG<br>R: GCAACACACAACATCTCCCA
Ssc-miR-125a-5p	TCCCTGAGACCCTTTAACCTGT
